# Northwest Texas Medical Association—Second Semi-Annual Meeting

**Published:** 1894-11

**Authors:** 


### Society Notes.

### The Northwest Texas Medical Association—Second Semi-Annual Meeting.

This Association was organized in Bowie some six months ago, a brief mention of which fact was made in the JOURNAL at the time, together with an announcement of the officers then elected.

The following is a summary of the proceedings of the second semi-annual meeting, held at Bowie, Bowie county, Texas, October 9th and 10th ult. The programmes sent out were quite pretentious, and reduced rates on railroads were secured, like a "sho nuff" medical association. It promises to be one of the largest, strongest and most popular local societies in the State. The JOURNAL rejoices to see such activity in organization, its hobby, and the one string upon which it has, for so many years kept twanging away. It will be seen that the citizens took a deep interest in the occasion and contributed to make it socially a brilliant success. They had an eye to business, meantime, and availed themselves of the opportunity to have a "trades display." The local paper gave a long account of the happening, emblazoned with startling headlines, and from that account, for which we are indebted to Secretary W. C. Dunaway, M. D., we extract the following minutes:

Meeting called to order by Chairman of Committee of Arrangements.

The following members answered to roll call: Drs. W. L. York, President, Decatur; B. Saunders, Fort Worth; H. Riley, Bowie; J. T. Sparkman, Alvord; W. C. Dunaway, Secretary, Bowie; C. C. Davis, Treasurer, Bowie; T. R. Allen, Greenwood;

B. F. Garrison, Chico; C. W. Parker, Alvord; T. H. Clark, Ryan, I. T.; J. F. Elliott, Bowie; W. D. Patton, Bowie; J. M. Stephens, Dan; J. F. Betram, Chico; J. L. Gaston, Bowie; J. D. Burch, Aurora; R. H. Mitchell, Bowie; B. C. Mitchell, Bowie; O. J. Kendall, Wichita Falls; W. B. Kyle, Leeter; W B. Palmer, Audubon; H. O. Stacy, Quanah; J. V. Clarke, Salona; J. R. Floyd, Paradise; E. A. Johnston, Henrietta; V. C. McNeese, Montague; H. F. Schoolfield, Sunset; W. A. Adams, Fort Worth; T. D. Thompson, Fort Worth; J. M. Gose, ———; J. S. Ross, Bowie; J. P. Clark, Bowie; Sneed Strong, Stoneburgh; Frank Gray, Fort Worth.

This being the second semi-annual meeting, and being temporarily organized, Dr. H. Riley, Chairman of the Committee of Arrangements, introduced Mayor J. H. Matthews, who, in a most appropriate way addressed the body and welcomed the visiting physicians to Bowie's hospitalities during the deliberations of the association.

President York was then introduced and responded in an appropriate speech.

Dr. York announced that the Association was ready for business, whereupon the Committee on Constitution and By-Laws were asked for a report.

The committee reported in full through its chairman, Dr. Saunders, after which it was read by section and so adopted as reported.

Dr. Saunders explained the importance of the judicial council, and insisted upon a full understanding of it by each member of the Association. It was also further discussed by Drs. B. C. Mitchell and J. L. Gaston.

The President then appointed the following gentlemen to serve on the judicial council, viz: Drs. Allen, Saunders, Mitchell, Sparkman, Davis and Patton.

The body then chose Dr. Saunders its president, and Dr. Clayton C. Davis its secretary.

Following is their report upon membership:

We, the judicial council, recommend the following for membership: Drs. W. B. Palmer, Audubon; H. O. Stacy, Quanah; O. J. Kendall, Wichita Falls; J. V. Clark, Salona; J. R. Floyd, Paradise; E. A. Johnson, Henrietta; W. B. Kyle, Leeter; J. C. McNeese, Montague; H. F. Schoolfield, Sunset; F. D. Thompson and W. A. Adams, Fort Worth.

The Association then adjourned to meet at the City Hall at 9 a. m., October 10th.

---

WEDNESDAY.—MORNING SESSION—9:15 A. M.

The Association met at 9:15 Wednesday morning, Dr. York in the chair.

Dr. Saunders moved that a committee be appointed to have printed 250 copies of the constitution and by-laws, and 250 blank applications for membership. Also that an assessment of $1 be made upon each member for defraying such expenses as might be incurred by the the Association. This publication committee consists of Drs. Dunaway and Davis, of Bowie.

The scientific work of the body was then resumed, whereupon Dr. Gaston, of Bowie, Chairman of Section of Practice of Medicine, read a very interesting and instructive paper upon "Continued Fevers," which was ably discussed by Drs. Sparkman, of Alvord, and Johnston, of Henrietta.

The regular proceedings were then suspended for the hearing of some interesting reports of cases by Dr. Mullens, of Fort Worth, upon eye, ear and throat.

Dr. Davis, of Bowie, then read a very interesting paper on hypnotics, which was thoroughly discussed by Drs. York, of Decatur; Sparkman, of Alvord, and Saunders, of Fort Worth.

Dr. Sparkman, of Alvord, presented a paper upon "Croupous Pneumonia" in children, which was fully appreciated by the Association, and ably discussed by Dr. York, of Decatur. Dr. Kendall, of Wichita Falls, then reported an unusual case of hyperpyrexia that recently occurred in his practice. Dr. Riley, of Bowie, also presented a clinical report of mild progressive senile gangrene produced from obstructions of emboli.

The President announced at this time that he had appointed Dr. J. C. McNeese, of Montague, Chairman of Section on Practice of Medicine; Dr. E. A. Johnson, of Henrietta, Chairman of Section on Obstetrics and Gynecology, and Dr. J. R. Flood, of Paradise, Chairman of Section on Surgery.

AFTERNOON SESSION.

Body called to order by President York. The judicial council then made the following report:

We, the judicial council, recommend the following gentlemen as worthy of membership in the N. W. T. M. Association, viz: Drs. J. M. Gose; J. S. Ross, J. P. Clark, Bowie; Sneed

Strong, Stoneburg, and Frank Gray, Fort Worth. Report received.

Dr. Riley then came forward as Chairman of Section on Surgery with a valuable paper, entitled "History of Surgery."

The report of a case and clinic was next introduced by Dr. C. W. Parker, of Alvord, as also report of operation for ovariotomy by Dr. York, of Decatur. The latter was discussed by Drs. Riley and Kendall.

Dr. Kendall reported a successful operation of "Super Pubic Cystotomy" for removal of stone in a child four years old.

Dr. Saunders, of Fort Worth, then offered a most valuable paper upon "Acute Intestinal Obstruction," making a powerful plea for early operations where the diagnosis was certain; and if certainty in diagnosis could not be reached, the exploratory incision was justifiable in doubtful cases. Fully discussed by Drs. York, Sparkman, Allen and B. C. Mitchell.

Dr. Allen, Chairman of Section on Gynecology, read a paper upon "Accidental Hemorrhage Before Labor."

Dr. W. D. Patton's paper upon the "Bleeding Uterus," was a masterly effort, and it brought out the effects upon that organ, of irritated sympathetic centers. Discussed by Drs. York, Davis and Johnson.

Dr. Frank Gray, of Fort Worth, read a very able paper on "Corneal Ulcers."

The following resolutions were unanimously adopted by the Association:

*Resolved*, That the N. W. T. M. Association tender a vote of thanks to Misses Mollie E. Jackson and Maggie Simmons and the forty young ladies of the city of Bowie, and to the Bowie string band for that most pleasing entertainment given the Northwest Texas Medical Association on Tuesday evening.

DR. RILEY.
DR. PATTON,
DR. DAVIS,
Committee.

*Resolved*, That the sincere thanks of this Association be offered the local physicians, citizens and especially the ladies of Bowie for their kind considerations of us while in their midst.

DR. SAUNDERS,
DR. YORK,
Committee.

After a short discussion of Dr. Dunaway's paper on Anæsthesia, and some short but timely talks by Drs. Saunders and York for the good of the Association, it adjourned to meet in the city of Bowie on the second Tuesday in March, 1895.

W. L. YORK, President.

W. C. DUNAWAY, Secretary.

THE BANQUET.

At the close of the meeting the physicians of the city tendered the Association a feast in the way of a banquet, which was spread in the Lowrie building, and it was elegant and temptingly prepared.

Dr. R. H. Mitchell, of that city, acted as master of ceremonies at the banquet, during the progress of which toasts were responded to as follows: "The Doctors' Wives," response by J. T. Roberts, of Bowie; "Texas Native Wines," by Dr. J. F. Elliott; "The Ladies of Bowie," by Dr. B. Saunders, Fort Worth; "North Texas Medical Association," by Dr. Round; "The Medical Profession, by Dr. J. R. Allen, of Greenwood; "The Doctors," by Dr. W. D. Patton, of Bowie; "Citizens of Bowie," by Dr. G. T. Sparkman, of Alvord; "Northwest Texas Association, 1894," by Dr. J. L. Gaston, of Bowie; "Relations of Clergymen to the Doctors," by Rev. W. A. Stuckey, of Bowie; "Christian Science," by Dr. B. C. Mitchell. Dr. Riley closed the speaking with a few well chosen remarks.

To the Bowie physicians, one and all, is due the credit of attracting this notable gathering to Bowie, and the selection of that city as the Association's permanent place of meeting. The occasion closed with a grand ball.

